# Life stressors and mental health: Depressive symptoms, anxiety, and suicidal ideation or intent during and after the COVID-19 pandemic

**DOI:** 10.1371/journal.pone.0340198

**Published:** 2026-02-11

**Authors:** Shakila Meshkat, Xiaoxuan Han, Qiaowei Lin, Vanessa K. Tassone, Reinhard Janssen-Aguilar, Wendy Lou, Shauna Major, Michael Cooper, Andrew Greenshaw, Venkat Bhat

**Affiliations:** 1 Interventional Psychiatry Program, St. Michael’s Hospital, Unity Health Toronto, Toronto, Ontario, Canada; 2 Department of Psychiatry, University of Alberta, Edmonton, Alberta, Canada; 3 Department of Biostatistics, Dalla Lana School of Public Health, University of Toronto, Toronto, Ontario, Canada; 4 Institute of Medical Science, Temerty Faculty of Medicine, University of Toronto, Toronto, Ontario, Canada; 5 Mental Health Research Canada, Toronto, Ontario, Canada; 6 Neuroscience and Mental Health Institute (NMHI), University of Alberta, Edmonton, Alberta, Canada; 7 Neuroscience Research Program, St. Michael’s Hospital, Toronto, Ontario, Canada; 8 Department of Psychiatry, University of Toronto, Toronto, Ontario, Canada; University of Eswatini, ESWATINI

## Abstract

**Objective:**

This study aimed to investigate the associations between life stressors and depressive symptoms, anxiety, and suicidal ideation or intent, while also examining how COVID-19 pandemic influenced these associations.

**Methods:**

This study used data from 19,950 participants in the Mental Health Research Canada cohort. Participants who were under the age of 20 or missing data for key mental health variables were excluded from the analyses. Primary outcomes included depressive symptoms, anxiety, and suicidal ideation or intent. Their associations with life stressors (concerns about catching COVID-19, economic downturn and inflation, job loss, challenges in paying household bills, and social factors) were analyzed using logistic regressions models.

**Results:**

Participants experiencing negative impacts from life stressors had significantly higher odds of having depressive symptoms (aOR: 1.46; 95% CI: 1.28–1.66), moderate to severe anxiety (aOR: 1.33; 95% CI: 1.15–1.53) and developing suicidal ideation or intent (aOR: 1.54; 95% CI: 1.36–1.74) compared to those experiencing positive impacts. The odds were higher after COVID‑19 than during COVID‑19 for depressive symptoms (aOR = 1.62 vs 1.29; p = 0.08) and moderate to severe anxiety (aOR = 1.52 vs 1.12; p = 0.03) among participants with negative impacts from life stressors. Participants who reported negative impacts from certain stressors had higher odds of adverse mental health outcomes. Challenges in paying household bills were most strongly associated with depressive symptoms (aOR: 1.96; 95% CI: 1.62–2.36), moderate to severe anxiety (aOR: 1.79; 95% CI: 1.45–2.21), and suicidal ideation or intent (aOR: 1.52; 95% CI: 1.28–1.82).

**Conclusions:**

This study highlights the significant impact of life stressors on mental health, with challenges related to paying household bills being a key factor linked to depressive symptoms, anxiety, and suicidal ideation or intent. The COVID-19 pandemic exacerbated these stressors, emphasizing the need for targeted mental health interventions. Findings are subject to limitations of cross-sectional, self-reported data and unvalidated suicidality measures.

## 1. Introduction

Stressful life events—ranging from acute disruptions like job loss or illness to ongoing challenges such as financial strain or caregiving responsibilities—are well-established risk factors for adverse mental and physical health outcomes [1–3] Even daily hassles such as commuting delays, interpersonal tensions, or administrative burdens can exert significant effects over time.³ These stressors have been linked to chronic illnesses (e.g., diabetes, asthma), academic difficulties, and increased service use [3,4]. Importantly, life stress is associated with heightened vulnerability to mental health conditions, including depression, anxiety, and suicidal ideation [1].

While severe traumatic events (e.g., assault or childhood abuse) have been extensively studied in relation to post-traumatic stress disorder (PTSD), less is known about the broader range of stressors that may precede common mental health symptoms in adults [5,6]. Some studies show strong associations between life stress and depression, anxiety, or suicidal thoughts [7–10], while others report weaker or inconsistent findings [8,9]. These discrepancies may reflect differences in measurement approaches, stressor types, or population characteristics. For anxiety in particular, both acute and chronic stressors are implicated in symptom onset and exacerbation, with the nature and intensity of stress exposure playing key roles [11–13]. Similarly, stress may contribute to suicidal ideation by intensifying feelings of hopelessness, a known risk factor for suicidal behavior. The COVID-19 pandemic introduced a global context of prolonged stress and uncertainty, affecting billions through lockdowns, economic instability, and social isolation. By 2023, over 7 million deaths had been reported globally [9], and symptoms of depression, anxiety, and suicidal ideation rose sharply [10,11]. Pandemic-related stressors have continued to affect individuals even after the acute public health threat subsided, with lingering effects including grief, financial insecurity, and unresolved trauma [12–16]. As such, understanding how life stressors relate to mental health remains a critical post-pandemic priority. This study aims to evaluate the associations between a wide range of life stressors—including both negative and positive perceptions—and symptoms of depression, anxiety, and suicidal ideation or intent. In addition, we explore how the COVID-19 pandemic may have influenced these associations.

## 2. Methods

### 2.1 Study design and data sources

This study was a cross-sectional investigation into the associations between life stressors and mental health outcomes, including the presence of depressive symptoms, anxiety, and suicidal ideation or intent among participants from the Mental Health Research Canada (MHRC) cohort, during and after the COVID-19 pandemic. Data for this study were collected by Pollara Strategic Insights through an online polling initiative between March 2020 and January 2024. While no formal probabilistic sampling strategy was employed, participants were recruited from an online panel of adult Canadians. The survey results were weighted by age, gender, and region to improve representativeness and align the weighted sample with the demographic composition of the Canadian adult population. The period from May 2020 to April 2023 was categorized as “during COVID-19”, and May 2023 to January 2024 as “after COVID-19”. This classification was based on the World Health Organization’s declaration ending the Public Health Emergency of International Concern (PHEIC) on May 5, 2023. The “after COVID-19” period reflects the most recent data available in the Mental Health Research Canada (MHRC) surveys at the time of analysis. Notably, MHRC did not collect data prior to March 2020, which is why the “during COVID-19” period begins in May 2020.

The MHRC polling data assessed the presence of depressive symptoms and levels of anxiety, and identified factors influencing mental health. This dataset provided insight into Canadians’ mental health and was accessible through the MHRC Data Hub: https://www.mhrc.ca/data-hub.To define the study population, while the dataset was primarily cross-sectional, it incorporated elements of a longitudinal design for some respondents. To ensure each individual was represented only once, we excluded participants with duplicate unique identifiers (UIDs) from Leger-supplied data (Supplier = 1) by retaining only their earliest survey response, following MHRC data cleaning recommendations. For records from other suppliers (Supplier = 2), duplicate UIDs were retained, as MHRC identified these as “false duplicates” (i.e., identical UIDs corresponding to different individuals). This approach ensured consistent and accurate handling of longitudinal responses. We also excluded those under the age of 20, and participants with missing data on key mental health outcomes (depressive symptoms, anxiety, suicidal ideation or intent). Therefore, the final inclusion criteria for the current analysis were: (1) completed participation in the MHRC survey between March 2020 and January 2024; (2) non-duplicate unique identifier; (3) age greater than 20 years; and (4) complete data on relevant life stressor exposures, mental health outcomes, and survey weights. Participants under age 20 were excluded to align with the study’s focus on adult mental health, as stressors and symptom presentations differ significantly from those in adolescents.

### 2.2 Exposure variable

Life stressors were defined based on 26 survey items evaluating participants’ perceptions of various challenges affecting their mental health. These items spanned a wide range of stressors, including concerns about COVID-19 infection (4 items), economic downturn and inflation (3 items), job-related stressors such as job loss or changes in employment (11 items), financial strain such as difficulty paying bills (2 items), and social/entertainment-related disruptions such as consuming news or using social media (6 items). Each item was rated on an 11-point scale from 0 (“very negative impact”) to 10 (“very positive impact”), with additional response options for “not applicable” and “don’t know/unsure”. Responses were grouped by stressor, and a stressor-specific average score was calculated for each participant. These averages were then dichotomized: scores <5 were classified as indicating a “negative impact,” and scores ≥5 as “positive impact”.

In addition to stressor-related indicators, an overall life stressor variable was created by averaging all 26 items for each participant and applying the same dichotomization threshold. Dichotomizing the 0–10 scores (<5 vs ≥ 5) allowed harmonized cross‑domain comparisons and mirrored MHRC’s negative-impact focus, while acknowledging the inevitable information loss relative to modeling the scales continuously [17,18].

### 2.3 Outcome variables

The outcome variables of the study include the presence of depressive symptoms, moderate to severe anxiety, and suicidal ideation or intent. Depressive symptoms were assessed using the nine-item Patient Health Questionnaire (PHQ-9) [19–21], which evaluates the frequency of having depressive symptoms over the past two weeks. Each item includes four response categories: “not at all,” “several days,” “more than half the days,” and “nearly every day,” corresponding to scores of 0, 1, 2, and 3, respectively. The severity of depressive symptoms (PHQ-9 scores) is determined by summing the scores across all nine items (total scores ranging from 0 to 27), with higher scores indicating more severe depressive symptoms. A PHQ-9 score of 10 or above is indicative of the presence of clinically meaningful depressive symptoms, whereas a score below 10 suggests the absence of depressive symptoms [19]. For analysis purposes, the presence or absence of depressive symptoms was coded as 1 and 0, respectively.

Anxiety was assessed using the Generalized Anxiety Disorder-7 (GAD-7) [22], which evaluates the frequency of having anxiety symptoms over the past 2 weeks. Each item included four response categories: “not at all,” “several days,” “more than half the days,” and “nearly every day,” corresponding to scores of 0, 1, 2, or 3, respectively [22]. The severity of anxiety (GAD-7 scores) is determined by summing the scores across all seven items, resulting in a total score ranging from 0 to 21, with higher scores indicating more severe anxiety. Scores of 15–21 indicate severe anxiety, 10–14 indicate moderate anxiety, 5–9 indicate mild anxiety, and 0–4 suggest minimal anxiety. For analysis purposes, the presence and absence of moderate to severe anxiety (GAD-7 score of 10 or higher) were coded as 1 and 0, respectively [22].

Suicidal ideation was measured through questions asking participants whether they had thought about suicide within the last 3 months, the last year, or had a previous history of suicidal thoughts. Suicidal intent was measured based on participants’ responses to whether they had made a plan to attempt suicide within the last 3 months, the last year, or had a history of planning a suicide attempt. Thus, participants were grouped into three categories: those reporting suicidal ideation, those reporting suicidal intent, and those preferring not to answer. For analysis purposes, suicidal ideation and suicidal intent were combined into a single binary outcome variable, where the presence of either suicidal ideation or intent, or a response of “prefer not to answer,” was coded as 1, and the absence of both ideation and intent was coded as 0. A separate sensitivity analysis was conducted for the suicidal ideation or intent outcome. In the primary analysis, participants who selected “prefer not to answer” were coded as having experienced suicidal ideation/intent (coded as 1). In the sensitivity analysis, we regrouped the outcome such that only those explicitly reporting suicidal ideation or intent were coded as 1, and those selecting “prefer not to answer” were grouped with those reporting no ideation/intent (coded as 0). Additionally, we performed multiple imputation (MI) to reduce potential bias from treating those responses as either all 1s or all 0s. Using the mice package, we imputed the suicidal ideation/intent variable (three categories: suicidal ideation/intent coded 1, none coded as 0, “prefer not to answer” coded as NA) with a polytomous logistic model, while keeping all exposure and covariate variables in the imputation model. Twenty datasets were created, where suicidal ideation/intent was then re-binarized (1 vs. 0) within each completed dataset and analyzed with the same survey-weighted multivariable logistic model used in the primary analysis. Estimates were pooled with Rubin’s rules. Both approaches allowed us to assess the impact of response uncertainty on the observed associations.

### 2.4 Covariates

Covariates included age, gender (man, woman or another gender identity), location of residence (urban or rural), household composition (living alone, living with others or other), parental status (having children aged 18 years or older, having children under 18 years, having children in both age categories, not being a parent or other), employment status (employed, unemployed, retired/student or other), highest level of education (elementary/high school, college/technical education, university or prefer not to say), household income (less than $20,000, $20,000–$49,999 or $50,000–$99,999), alcohol use (never, monthly or less, 2–4 times a month, 2–3 times a week or 4 or more times a week), and cannabis use (never, monthly or less, 2–4 times a month, 2–3 times a week or 4 or more times a week). Covariates were selected based on their potential impacts on mental health outcomes.

### 2.5 Statistical analyses

Descriptive statistics were performed to summarize the characteristics of the study population. Categorical variables were presented as weighted percentages with raw frequency, and continuous variables were reported as weighted means with standard deviations. Weighted Chi-square tests were used to assess differences between groups for categorical variables, and weighted t-tests were employed to assess continuous variables. Multivariable logistic regression models were used to examine the associations between overall life stressors and specific stressors (i.e., concerns about catching COVID-19, economic downturn and inflation, job loss, challenges in paying household bills, and social factors) with mental health outcomes, including depressive symptoms, anxiety, and suicidal ideation or intent. All models were adjusted for a pre-specified covariate set based on prior literature and clinical significance; no stepwise screening was performed. Separate models were fitted to examine the associations between life stressors and the mental health outcomes. As a sensitivity analysis, we conducted both unadjusted and adjusted linear regression analyses to examine the associations between life stressors and PHQ-9 and GAD-7 scores, treating these outcomes as continuous variables. These models assessed whether symptom severity varied by exposure, providing complementary evidence to the primary binary outcome models. This allowed us to assess the robustness of the findings across different outcome specifications.

We also conducted separate analyses to assess these associations during and after COVID-19, using Z-tests to compare changes over time. All analyses accounted for the complex survey design and sampling weights using the survey package in R (version 4.3.2). A survey design object was created using the svydesign() function, specifying the weights provided by MHRC. Weighted logistic and linear regression models were then fitted using the svyglm() function, with the family argument set to binomial for logistic models and gaussian for linear models. This approach ensured valid variance estimation and generalizability to the broader Canadian adult population. Results from the linear regression models were reported as coefficient estimates (Coef.) and adjusted coefficient estimates (aCoef.) with 95% confidence intervals (CI). Results from the logistic regression models were reported as odds ratios (OR) and adjusted odds ratios (aOR) with 95% CI. The aCoefs and aORs were derived from models adjusted for all selected covariates. Statistical significance was assessed using a two-sided p-value < 0.05, as a significance level of p-value < 0.05 was used consistently for both bivariate and multivariable analyses, in line with common practice in epidemiological research. Given the number of outcomes, stressor categories, and time-period-specific models, there is an increased risk of type I error. Each fully‑adjusted model contained five specific life stressors, which formed a single “family” of related hypotheses. To guard against inflation of the family‑wise type‑I error while retaining more power than a Bonferroni split, we applied the Holm step‑down procedure to the five p‑values. Holm controls the family‑wise error rate but, unlike Bonferroni, adjusts less stringently for the strongest signals and therefore maintains better sensitivity when true associations are expected. Results are interpreted as statistically significant only when the Holm‑adjusted p‑value is < 0.05 among the five specific life stressors.

Missing data were handled using listwise deletion within the svyglm() function. Participants with missing exposure, outcome, or survey weight information were excluded from the analysis. Covariates with partial missingness were retained in model specification. We assessed the extent of missingness for all model variables and found that all covariates had ≤ 7.2% missingness, while exposure and outcome variables had no missing values. Thus, the covariate-level missingness was low, and no missingness was present in key exposure or outcome variables. We assessed potential selection bias by re‑fitting simple survey‑weighted logistic models that included only the overall life stressors variable (no covariates) separately in the included and excluded groups. We then compared the resulting OR.

Model assumptions were assessed for all regression analyses. Multicollinearity was evaluated using variance inflation factors (VIFs), and no covariates exceeded a VIF of 5, indicating acceptable levels of collinearity. For linear regression models used in sensitivity analyses, normality of residuals was assessed using Q-Q plots and histograms, and assumptions were deemed to be reasonably met. We compared the parsimony of all survey‑weighted logistic‑regression models using the Akaike Information Criterion (AIC).

### 2.6 Ethical considerations

Ethical clearance was granted by Health Canada Research Ethics Board. MHRC also obtained written informed consent from the study participants prior to administration of the questionnaire. Pollara Strategic Insights is a founding, accredited Gold Seal member of the Canadian Research Insights Council (CRIC). MHRC is in full compliance with the CRIC Canadian Code of Market, Opinion, and Social Research and Data Analytics, the CRIC Public Opinion Research Standards and Disclosure Requirements, the CRIC Pledge to Canadians, and ISO 20252:2019. Pollara’s Privacy Policy affirms and details our commitment to protecting privacy.

## 3. Results

### 3.1 Descriptive statistics

**Fig 1:**
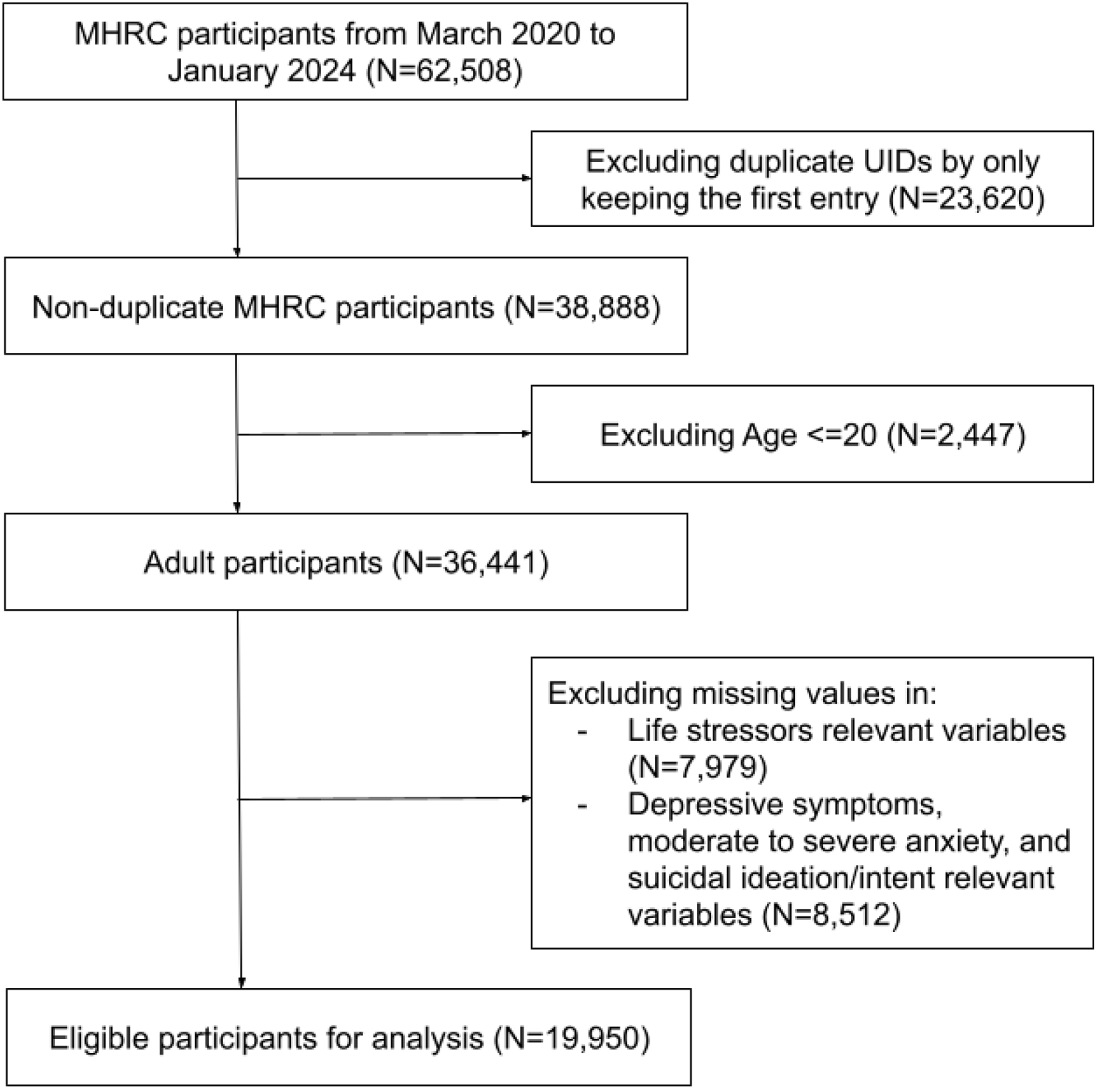
Study participant inclusion flowchart.

A total of 19,950 participants were selected for the study by excluding duplicated unique identifiers (N = 23,620), participants under the age of 20 (N = 2,447), and those with missing data for the exposure (N = 7,979) and key mental health outcomes, including depressive symptoms, moderate to severe anxiety, and suicidal ideation or intent (N = 8,512) (Fig 1). Specifically, 14,940 participants were categorized into the “during COVID-19” period and 4,991 participants into the “after COVID-19” period, with the remaining participants falling outside these two periods, based on their survey response time.

**Fig 2:**
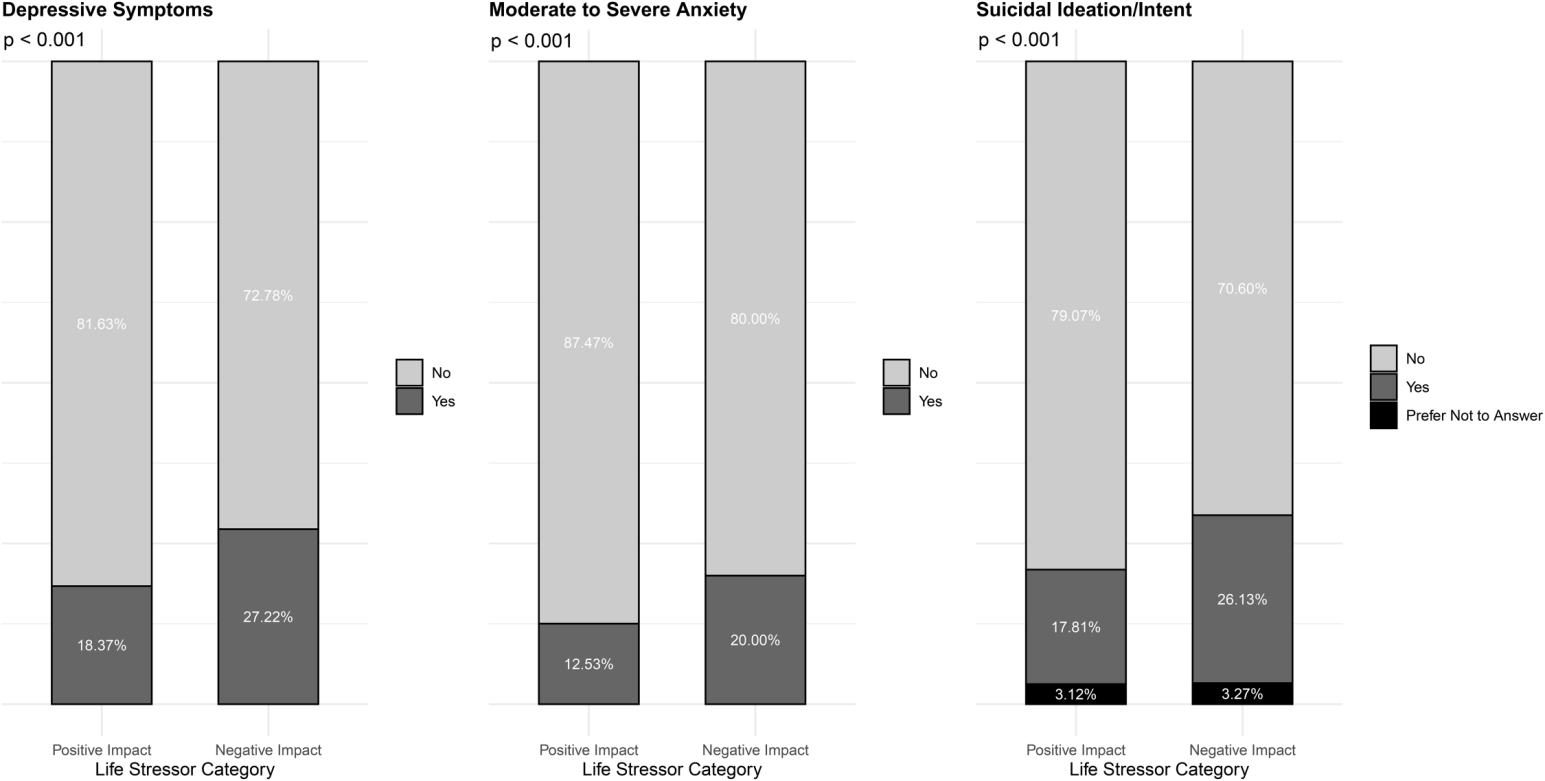
Prevalence of mental health outcomes by life stressor category.

The prevalence of depressive symptoms, moderate to severe anxiety, and suicidal ideation or intent varied significantly by the reported impact of life stressors (Fig 2). Among participants who experienced negative impacts from life stressors, 27.22% had depressive symptoms compared to 18.37% among those who reported positive impacts. Similarly, moderate to severe anxiety was more prevalent in the negative impact group (20.00%) than in the positive impact group (12.53%). Suicidal ideation or intent was reported by 26.13% of participants experiencing negative impacts, contrasting with 17.81% in the positive impact group.

Table 1 presents the descriptive characteristics of participants stratified by the presence of depressive symptoms, moderate to severe anxiety, and suicidal ideation or intent, with weighted percentages. Overall, participants with depressive symptoms, moderate to severe anxiety, and suicidal ideation or intent were more likely to report negative impacts from life stressors. Specifically, during COVID-19, participants who experienced negative life stressors showed significantly higher rates of depressive symptoms (76.6%) and moderate to severe anxiety (78.3%) compared to those who experienced positive impacts. After COVID-19, the trend remained, with 75.8% of participants with depressive symptoms and 76.2% with moderate to severe anxiety reporting negative life stressors (S1 Table).

**Table 1:** Characteristics of the study sample.

Variables	Total	Depressive Symptoms No	Depressive Symptoms Yes	*p-value*	Moderate to Severe Anxiety No	Moderate to Severe Anxiety Yes	*p-value*	Suicidal Intent or Ideation No	Suicidal Intent or Ideation Yes	Suicidal Ideation or Intent Prefer Not to Answer	*p-value*
**Sample Size**	19,950	14,874	5,076	–	16,243	3,707		14,381	4,919	650	
**Age (Mean (SD))**	47.28 (16.7)	49.51 (16.**7**)	40.74 (14.8)	<0.001	48.97 (16.7)	39.89 (14.3)	<0.001	49.56 (16.6)	41.37 (15.3)	41.61 (15.3)	<0.001
**Gender (%)**				<0.001			<0.001				0.135
Man	8,852 (43.2)	6,940 (44.4)	1,912 (39.3)		7,569 (44.6)	1,283 (36.8)		6,465 (42.8)	2,099 (44.3)	288 (45.6)	
Woman	10,997 (56.8)	7,881 (55.6)	3,116 (60.7)		8,617 (55.4)	2,380 (63.2)		7,875 (57.2)	2,765 (55.7)	357 (54.4)	
**Rural (%)**				<0.001			0.006				0.079
Urban	17,259 (87.9)	12,781 (87.3)	4,478 (89.6)		13,997 (87.6)	3,262 (89.4)		12,368 (87.6)	4,310 (88.3)	581 (90.7)	
Rural	2,691 (12.1)	2,093 (12.7)	598 (10.4)		2,246 (12.4)	445 (10.6)		2,013 (12.4)	609 (11.7)	69 (9.3)	
**Household Composition (%)**				0.001			0.693				<0.001
Live Alone	3,627 (19.2)	2,613 (18.6)	1,014 (21.0)		2,959 (19.2)	668 (19.3)		2,443 (18.0)	1,050 (22.5)	134 (20.8)	
Live with Others	15,961 (79.0)	12,014 (79.7)	3,947 (76.8)		13,003 (79.1)	2,958 (78.7)		11,699 (80.3)	3,772 (75.5)	490 (75.5)	
Other	362 (1.8)	247 (1.7)	115 (2.2)		281 (1.8)	81 (2.0)		239 (1.7)	97 (2.0)	26 (3.7)	
**Parental Status (%)**				<0.001			<0.001				<0.001
Has Children >= 18	2,843 (14.4)	2,156 (14.8)	687 (13.1)		2,339 (14.8)	504 (12.9)		2,166 (15.4)	608 (11.8)	69 (10.3)	
Has Children >= 18 and < 18	666 (3.2)	479 (3.0)	187 (3.7)		520 (3.0)	146 (3.9)		458 (3.0)	182 (3.4)	26 (4.0)	
Has Children < 18	4,190 (19.8)	2,958 (18.5)	1,232 (23.9)		3,239 (18.6)	951 (25.4)		2,915 (18.9)	1,140 (22.5)	135 (20.9)	
Not a Parent	11,737 (59.9)	8,886 (60.8)	2,851 (57.0)		9,725 (60.9)	2,012 (55.4)		8,475 (60.0)	2,884 (60.1)	378 (57.8)	
Other	514 (2.7)	395 (2.8)	119 (2.3)		420 (2.7)	94 (2.6)		367 (2.6)	105 (2.2)	42 (6.9)	
**Employment Status (%)**				<0.001			<0.001				<0.001
Employed	12,330 (59.3)	8,966 (57.5)	3,364 (64.8)		9,807 (57.7)	2,523 (66.6)		8,616 (57.1)	3,296 (65.6)	418 (63.3)	
Retired/Student	5,564 (30.7)	4,656 (34.4)	908 (19.3)		4,995 (33.7)	569 16.3)		4,518 (34.5)	935 (20.7)	111 (17.0)	
Unemployed	605 (3.0)	346 (2.3)	259 (5.1)		411 (2.4)	194 (5.4)		348 (2.3)	219 (4.7)	38 (5.1)	
Other	1,451 (7.0)	906 (5.8)	545 (10.8)		1,030 (6.1)	421 (11.6)		899 (6.1)	469 (9.1)	83 (14.6)	
**Highest Level of Education**				<0.001			0.001				0.003
College/Technical	6,577 (33.2)	4,924 (33.5)	1,653 (32.6)		5,387 (33.5)	1,190 (32.0)		4,755 (33.2)	1,632 (34.0)	190 (29.8)	
Elementary/High School	3,919 (20.2)	2,772 (19.3)	1,147 (22.9)		3,088 (19.6)	831 (22.7)		2,712 (19.6)	1,056 (21.4)	151 (24.8)	
University	9,334 (46.6)	7,088 (47.2)	2,246 (44.6)		7,673 (46.8)	1,661 (45.4)		6,854 (47.2)	2,205 (44.7)	275 (45.5)	
**Household Income**				<0.001			<0.001				<0.001
< $20,000	1,155 (6.1)	650 (4.8)	505 (10.1)		775 (5.1)	380 (10.7)		666 (5.0)	422 (8.8)	67 (12.5)	
$100,000 or more	5,960 (31.8)	4,755 (33.9)	1,205 (25.5)		5,014 (32.8)	946 (27.4)		4,583 (33.8)	1,242 (26.6)	135 (25.8)	
$20,000–$49,999	4,474 (24.9)	3,100 (23.5)	1,374 (29.3)		3,550 (24.2)	974 (28.1)		3,004 (23.6)	1,333 (28.7)	137 (27.1)	
$50,000–$99,999	6,904 (37.1)	5,218 (37.8)	1,686 (35.1)		5,710 (37.9)	1,194 (33.7)		5,027 (37.6)	1,689 (35.9)	188 (34.6)	
**Alcohol Use**				<0.001			<0.001				<0.001
Never	2,010 (21.0)	1,462 (20.5)	548 (22.3)		1,583 (20.4)	427 (23.5)		1,362 (20.5)	544 (20.8)	104 (31.3)	
Monthly or Less	2,976 (30.6)	2,105 (29.2)	871 (35.0)		2,361 (30.2)	615 (32.7)		2,012 (29.9)	841 (32.0)	123 (34.4)	
2–4 times/month	2,096 (21.8)	1,552 (21.7)	544 (21.9)		1,684 (21.7)	412 (22.3)		1,470 (21.7)	565 (22.7)	61 (17.2)	
2–3 times/week	1,520 (16.1)	1,215 (17.6)	305 (11.7)		1,280 (17.0)	240 (12.2)		1,107 (17.1)	373 (14.3)	40 (10.3)	
4 or more times/week	949 (10.5)	732 (11.0)	217 (9.1)		786 (10.8)	163 (9.3)		731 (10.8)	249 (10.1)	22 (6.8)	
**Cannabis Use**				<0.001			<0.001				<0.001
Never	7,013 (74.1)	5,579 (79.5)	1,434 (58.2)		6,023 (77.7)	1,067 (58.7)		5,291 (80.5)	1,461 (56.4)	261 (75.1)	
Monthly or Less	1,156 (11.7)	739 (10.1)	417 (16.7)		850 (11.0)	291 (15.2)		674 (9.8)	442 (17.4)	40 (10.6)	
2–4 times/month	409 (4.4)	228 (3.3)	181 (7.6)		283 (3.7)	137 (7.6)		203 (3.1)	201 (8.4)	5 (1.1)	
2–3 times/week	301 (3.0)	163 (2.2)	138 (5.1)		187 (2.4)	107 (5.3)		142 (2.0)	150 (5.6)	9 (2.5)	
4 or more times/week	672 (6.8)	357 (4.9)	315 (12.4)		412 (5.3)	255 (13.3)		319 (4.6)	318 (12.2)	35 (10.7)	
**Life Stressors (%)**				<0.001			<0.001				<0.001
Positive Impact	6,184 (31.4)	4,972 (34.0)	1,212 (23.6)		5,327 (33.4)	857 (22.3)		4,800 (33.9)	1,185 (23.8)	199 (30.6)	
Negative Impact	13,766 (68.6)	9,902 (66.0)	3,864 (76.4)		10,916 (66.6)	2,850 (77.7)		9,581 (66.1)	3,734 (76.2)	451 (69.4)	

*P-values were calculated by weighted Chi-square tests for categorical variables, and weighted t-tests for continuous variables. Categorical characteristics were reported as raw frequency and weighted percent. Continuous characteristics were reported as weighted mean and standard deviation. The empty cells on the row of ”Positive Impact“ indicates the reference category.

### 3.2 Association between life stressors and depressive symptoms

In the multivariable logistic regression model, participants experiencing negative impacts from life stressors had higher odds of having depressive symptoms (aOR: 1.46; 95% CI: 1.28–1.66; p < 0.001) compared to those experiencing positive impacts. In the multivariable logistic regression model with the inclusion of specific stressors, participants experiencing negative impacts from stressors of concerns from challenges in paying household bills (aOR: 1.96; 95% CI: 1.62–2.36; p-value adjusted < 0.001) had higher odds of having depressive symptoms (Table 2).

**Table 2:** Associations of specific stressors with depressive symptoms, moderate to severe anxiety, and suicidal ideation/intent during March 2020 – Jan 2024.

Life Stressor Types		Depressive Symptoms		Moderate to Severe Anxiety		Suicidal Ideation/Intent	
		aOR. (95% CI)	p-value (p-value adjusted)	aOR. (95% CI)	p-value (p-value adjusted)	aOR. (95% CI)	p-value (p-value adjusted)
Concerns about Catching COVID-19	Positive Impact						
	Negative Impact	1.25 (0.98, 1.59)	0.0021 (0.2)	1.34 (1.02, 1.76)	0.036 (0.13)	1.40 (1.12, 1.74)	0.0025 (0.0078)
Economic Downturn/Inflation	Positive Impact						
	Negative Impact	1.16 (1.01, 1.34)	0.035 (0.14)	1.18 (1.01, 1.37)	0.032 (0.13)	1.28 (1.12, 1.47)	< 0.001
Job Loss	Positive Impact						
	Negative Impact	0.88 (0.71, 1.07)	0.20 (0.40)	0.84 (0.67, 1.05)	0.13 (0.25)	0.88 (0.72, 1.07)	0.19 (0.37)
Challenges in Paying Household Bills	Positive Impact						
	Negative Impact	1.96 (1.62, 2.36)	<0.001	1.79 (1.45, 2.21)	<0.001	1.52 (1.28, 1.82)	< 0.001
Social Factors	Positive Impact						
	Negative Impact	1.02 (0.80, 1.30)	0.36 (0.41)	1.32 (0.96, 1.82)	0.085 (0.025)	1.00 (0.77, 1.31)	0.98 (0.37)

In separate analyses, participants experiencing negative impacts from life stressors had higher odds of depressive symptoms after COVID-19 (aOR = 1.62) than during COVID-19 (aOR = 1.29), although the evidence for this difference was modest (p = 0.08) (Table 3). For specific stressors, the aOR for challenges paying household bills was lower after COVID‑19 (1.05) than during COVID‑19 (1.85; p < 0.001), and for social factors it was lower after COVID‑19 (1.16) than during COVID‑19 (1.64; p = 0.021). In contrast, the aOR for job loss was higher after COVID‑19 (1.68) than during COVID‑19 (1.02; p = 0.06), and for economic downturn/inflation it was slightly higher after COVID‑19 (1.20) than during COVID‑19 (1.11; p = 0.85), with weak evidence for this difference (Table 4).

**Table 3:** Associations of life stressors with depressive symptoms and moderate to severe anxiety.

Time	Life Stressors	Depressive Symptoms			Moderate to Severe Anxiety		
		aOR (95% CI)	*p-value*	*p-value* *(Z-test)*	aOR (95% CI)	*p-value*	*p-value (Z-test)*
March 2020 – Jan 2024	Positive Impact						
	Negative Impact	1.46 (1.28, 1.66)	<0.001	–	1.33 (1.15, 1.53)	<0.001	
During Covid	Positive Impact			0.08			0.03
	Negative Impact	1.29 (1.05, 1.59)	0.02		1.12 (0.89, 1.39)	0.3	
After Covid	Positive Impact						
	Negative Impact	1.62 (1.37, 1.92)	<0.001		1.52 (1.26, 1.82)	<0.001	

**Table 4:** Associations of specific stressors with depressive symptoms and moderate to severe anxiety during and after COVID-19.

Life Stressor Types	Time	Impact	Depressive Symptoms			Moderate to Severe Anxiety		
			aOR. (95% CI)	p-value (p-value adjusted)	p-value (Z-test)	aOR. (95% CI)	p-value (p-value adjusted)	p-value (Z-test)
Concerns about Catching COVID-19	During COVID-19	Positive Impact						
		Negative Impact	1.27 (1.13, 1.42)	< 0.001 (< 0.001)		1.46 (1.19, 1.79)	< 0.001 (< 0.001)	
	After COVID-19	Positive Impact						
		Negative Impact	–					
Economic Downturn/Inflation	During COVID-19	Positive Impact			0.85			0.98
		Negative Impact	1.11 (0.99, 1.24)	0.066 (0.013)		1.20 (1.05, 1.36)	0.0056 (0.011)	
	After COVID-19	Positive Impact						
		Negative Impact	1.20 (1.01, 1.43)	0.036 (0.11)		1.28 (1.06, 1.55)	0.0094 (0.029)	
Job Loss	During COVID-19	Positive Impact			0.06			0.0053
		Negative Impact	1.02 (0.89, 1.16)	0.78 (0.83)		0.99 (0.85, 1.15)	0.90 (0.95)	
	After COVID-19	Positive Impact						
		Negative Impact	1.68 (1.07, 2.66)	0.024 (0.10)		2.29 (1.36, 3.87)	0.0019 (0.0077)	
Challenges in Paying Household Bills	During COVID-19	Positive Impact			< 0.001			0.069
		Negative Impact	1.85 (1.54, 2.22)	< 0.001 (< 0.001)		1.23 (1.01, 1.50)	< 0.001 (< 0.001)	
	After COVID-19	Positive Impact						
		Negative Impact	1.05 (0.66, 1.67)	0.83 ***(0.85)***		0.69 (0.40, 1.17)	0.17 (0.19)	
Social Factors	During COVID-19	Positive Impact			0.021			0.21
		Negative Impact	1.64 (1.45, 1.85)	< 0.001 (< 0.001)		1.61 (1.41, 1.85)	< 0.001 (< 0.001)	
	After COVID-19	Positive Impact						
		Negative Impact	1.16 (0.87, 1.55)	0.32 (0.63)		1.33 (0.96, 1.83)	0.085 (0.19)	

In the sensitivity analysis of treating depressive symptoms continuously (S2 Table), the association between life stressors and depressive symptoms remained consistent. During the full study period (March 2020 to January 2024), participants experiencing negative life stressors had higher adjusted depressive symptom severity (total PHQ-9 scores) (aCoef.: 1.12; 95% CI: 0.84–1.40; p < 0.001). The aCoef was higher after COVID‑19 (1.56) than during COVID‑19 (0.67; p = 0.002), indicating a stronger association between negative life stressors and depressive symptom severity over time (p = 0.002).

### 3.3 Association between life stressors and moderate to severe anxiety

In the multivariable logistic regression model, participants experiencing negative impacts from life stressors had significantly higher odds of having moderate to severe anxiety (aOR: 1.33; 95% CI: 1.15–1.53; p < 0.001) compared to those experiencing positive impacts. In the multivariable logistic regression model with the inclusion of specific stressors, participants experiencing negative impacts from challenges in paying household bills (aOR: 1.79; 95% CI: 1.45–2.21; p-value adjusted < 0.001) had higher odds of having moderate to severe anxiety (Table 2).

In separate analyses, participants experiencing negative life stressors had higher odds of moderate to severe anxiety after COVID‑19 (aOR = 1.52) than during COVID‑19 (aOR = 1.12; p = 0.03) (Table 3). For specific stressors, the aOR for challenges paying household bills was lower after COVID‑19 (0.69) than during COVID‑19 (1.23; p = 0.069), and for social factors it was lower after COVID‑19 (1.33) than during COVID‑19 (1.61; p = 0.21). In contrast, the aOR for job loss was higher after COVID‑19 (2.29) than during COVID‑19 (0.99; p = 0.0053), and for economic downturn/inflation it was slightly higher after COVID‑19 (1.28) than during COVID‑19 (1.20; p = 0.98), with weak evidence for this difference (Table 4).

In the sensitivity analysis treating moderate to severe anxiety continuously (S2 Table), the association between negative life stressors and moderate to severe anxiety remained consistent. During the full study period (March 2020 to January 2024), participants experiencing negative life stressors had higher anxiety severity (total GAD-7 scores) (aCoef.: 0.94; 95% CI: 0.71–1.17; p < 0.001). The association was stronger after COVID‑19 (aCoef = 1.29) than during COVID‑19 (aCoef = 0.59; p = 0.004), indicating a stronger association between negative life stressors and developing anxiety over time (p = 0.004).

### 3.4 Association between life stressors and suicidal ideation or intent

In the multivariable logistic regression model, participants experiencing negative impacts from life stressors had higher odds of developing suicidal ideation or intent (aOR: 1.54; 95% CI: 1.36–1.74; p < 0.001) compared to those experiencing positive impacts (Table 5). In the multivariable logistic regression model with the inclusion of specific stressors, participants experiencing negative impacts from concerns about catching COVID-19 (aOR: 1.40; 95% CI: 1.12–1.74; p-value adjusted = 0.0078), economic downturn/inflation (aOR: 1.28; 95% CI: 1.12–1.47; p-value adjusted < 0.001), and challenges in paying household bills (aOR: 1.52; 95% CI: 1.28–1.82; p-value adjusted < 0.001) had higher odds of having suicidal ideation or intent (Table 2). Sensitivity analysis yielded consistent results for overall life stressors, with an aOR of 1.67 (95% CI: 1.47–1.90; p < 0.001) (S4 Table). In the MI analysis that treated “prefer not to answer” as missing and imputed using polytomous regression, the association between negative life stressors and suicidal ideation/intent remained virtually unchanged (pooled aOR ≈ 1.50; 95% CI: 1.37–1.64) (Table 5). Thus, our findings are robust to alternative handling of the ambiguous response category “prefer not to answer”.

**Table 5:** Associations between life stressors and suicidal ideation or intent.

	Time	Life Stressors	Suicidal Intent or Ideation	
			aOR (95% CI)	p-value
Main Analysis	March 2020 - Jan 2024 (n = 19,706)	Positive Impact		
		Negative Impact	1.54 (1.36, 1.74)	<0.001
Sensitivity Analysis (Regrouped “prefer not to answer”)	March 2020 - Jan 2024 (n = 19,706)	Positive Impact		
		Negative Impact	1.67 (1.47, 1.90)	<0.001
Sensitivity Analysis (Multiple Imputation)	March 2020 - Jan 2024 (n = 19,706)	Positive Impact		
		Negative Impact	1.50 (1.37, 1.64)	<0.001

### 3.5 Model diagnostics and bias check

AIC values ranged from 4,405–9,612 across the nine models examined. The lowest AIC was observed for the “After‑COVID” moderate‑to‑severe‑anxiety model (AIC = 4,405), suggesting this specification achieved the most efficient fit. The relative ordering of AICs did not alter the substantive direction or significance of any association reported above (S3 Table).

Of 19,950 respondents, 18,343 (92.0%) had complete data and were included; 1,607 (8.0%) were excluded because ≥1 covariate was missing. We re‑estimated the overall life stressor associations in both subsets using identical reduced models (overall life stressors only). ORs for negative vs. positive impact were very similar in included vs. excluded participants: depressive symptoms 1.21 (1.06–1.39) vs. 1.33 (0.83–2.16); moderate to severe anxiety 1.27 (1.11–1.46) vs. 1.59 (1.00–2.51); suicidal ideation/intent 1.58 (1.46–1.72) vs. 1.49 (1.12–1.96) (S4 Table). These consistent estimates indicate minimal selection bias from listwise deletion and support the robustness of our results.

## 4. Discussion

In this study of MHRC polling (March 2020–January 2024), respondents who rated overall life stressors as having a negative impact had higher odds of depressive symptoms, moderate‑to‑severe anxiety, and suicidal ideation/intent than those reporting positive impact. Among specific stressors, difficulty paying household bills remained significant after Holm adjustment across all three outcomes. Concerns about catching COVID‑19 remained significant only for suicidal ideation/intent; its associations with depression and anxiety did not survive Holm adjustment. For economic downturn/inflation, the pooled association with suicidal ideation/intent was positive. Overall associations were stronger after May 2023 than May 2020–April 2023. In cross‑sectional, self‑report polling, reverse causation (current symptoms shaping how stressors are perceived) and common‑method variance can inflate associations, and unmeasured confounding (e.g., prior diagnoses, social support) may account for part of the observed links.

The results of our study are in line with previous research that has indicated the positive association between negative life stress and depression, anxiety and suicide [23,24]. Currier et al. (2016) used national Australian data to examine the relationship between life stressors and mental health outcomes, finding that individuals experiencing significant life stress were at a higher risk for developing depression and anxiety, with a strong association between co-occurring life stress and depression [23]. Furthermore, Sun et al. (2017) conducted a similar study among university students in China which also explored the impact of life events, coping strategies, and depression severity [24]. Their findings support our results, as negative life events were found to significantly contribute to the likelihood of depression, with a stronger association observed in the progression from non-depression to mild depression. In another study Zou et al., (2018) [25] found that negative life events were positively correlated with mental health problems, similar to the findings in our study. Zou et al. (2018) highlighted that different types of negative life events had distinct associations with specific mental health issues, such as depression, anxiety, and stress [25]. Notably, in this study, participants who experienced more “interpersonal relationship” issues were more likely to report anxiety and stress, whereas those facing “change for adaptation” related problems were more likely to experience depression and anxiety. This focus on specific types of life events was not assessed by the MHRC, which only classified positive and negative life events.

Negative life stressors significantly influence mental health outcomes through complex and multifaceted mechanisms. One key pathway involves dysregulation of the hypothalamic-pituitary-adrenal (HPA) axis, leading to prolonged elevations in cortisol levels, which can impair neurogenesis and structural integrity in brain regions such as the hippocampus and prefrontal cortex—regions critical for emotional regulation and cognitive processing [26]. Chronic stress is also associated with heightened activity in the amygdala, reinforcing fear and threat perceptions while diminishing the brain’s capacity to process positive experiences [27]. At the neuroinflammatory level, stress can induce systemic inflammation, marked by elevated pro-inflammatory cytokines like Interleukin-6 and Tumor necrosis factor-α, which have been implicated in the pathophysiology of depression and anxiety [28]. Furthermore, stress-related oxidative stress contributes to cellular damage, exacerbating neurological vulnerability [28,29]. Psychologically, negative life events challenge an individual’s coping mechanisms, potentially fostering maladaptive responses such as rumination and avoidance behaviors, which perpetuate negative affect and impede recovery [29]. However, our cross‑sectional, observational design cannot test mechanistic pathways; these explanations are provided as context rather than causal proof.

The results of our study indicated that suicidal ideation or intent exhibited the strongest association with negative life stressors, followed by depressive symptoms and anxiety. The strong association between negative life stressors and suicidal ideation in our study can be due to the stress-diathesis model, which highlights the interplay between predisposing factors (diathesis) and external stressors [30]. Life stressors, such as significant negative events, can act as triggers for suicidal thoughts and behaviors in individuals with a predisposition (diathesis) that includes factors like mental illness, substance abuse, aggression, and hopelessness [30]. While life stressors alone do not necessarily lead to suicidal ideation, they can significantly increase the likelihood when combined with existing vulnerabilities [30,31]. Our findings align with this model, demonstrating that individuals facing negative life events are more likely to experience depressive symptoms and anxiety, but the escalation to suicidal ideation reflects a more severe outcome, indicating the combined effect of stress and underlying mental health issues.

Compared with the period *during* the pandemic, the *overall* association between negative life stressors and mental‑health outcomes appeared stronger *after* May 2023 (depression aOR 1.62 vs 1.29, Z‑test p = 0.08; anxiety 1.52 vs 1.12, p = 0.03). This pattern may reflect evolving macro‑economic and service‑access conditions rather than pandemic status alone. Several studies have indicated the negative impact of COVID-19 pandemic on mental health [32]. This may be attributed to prolonged uncertainty, social isolation, economic instability, and health concerns, all of which likely acted as significant stressors and contributed to worsened mental health outcomes [32]. The pandemic not only introduced new stressors but also intensified existing vulnerabilities, leading to increased psychological distress. Disruptions to daily routines, limited access to social support, and challenges in obtaining mental health care added to the difficulties faced by many [32].

The findings from this study highlighted the significant impact of financial stress, particularly challenges in paying household bills, on mental health outcomes of depression, anxiety, and suicidal ideation, with the highest odds observed for this particular stressor. Pooled aORs for challenges in paying household bills were 1.96 (depressive symptoms), 1.79 (anxiety), and 1.52 (suicidal ideation or intent), each remaining significant after adjustment. This is consistent with previous research, which has established a strong link between financial strain and poorer mental health outcomes. For instance, Kuehner (2017) emphasized the connection between economic stressors, such as job loss and financial hardship, and an increased risk of depression and anxiety [33]. Furthermore, financial strain has been recognized as a critical factor contributing to suicidal ideation, particularly in times of economic downturn [34]. In our study, concerns about catching COVID‑19 were significantly associated only with suicidal ideation or intent (aOR 1.40, 95% CI 1.12–1.74, Holm p = 0.0078); associations with depression (aOR 1.25, 95% CI 0.98–1.59) and anxiety (aOR 1.34, 95% CI 1.02–1.76) did not remain significant after Holm adjustment. The psychological effects of the COVID-19 pandemic, particularly anxiety due to health concerns and uncertainty, have been widely documented [35], reinforcing the relevance of these stressors in the current context. For suicidal ideation or intent, economic downturn/inflation was also associated (aOR 1.28, 95% CI 1.12–1.47), aligning with studies that show how economic crises can trigger feelings of hopelessness and increase the risk of suicide [34].

Notably, the associations between life stressors and mental health outcomes varied across the three time frames—during COVID-19, after COVID-19, and across the full study period—highlighting the temporal dynamics of stress and psychological distress. During the COVID-19 period, life stressors were significantly associated with increased odds of depressive symptoms and moderate to severe anxiety; however, the aORs were slightly attenuated compared to the post-COVID period. Specifically, the adjusted associations between negative stressor impact and depressive symptoms strengthened after the pandemic (aOR = 1.62) relative to the COVID-19 period (aOR = 1.29), with similar patterns observed for anxiety outcomes (aOR = 1.52 post-COVID vs. 1.12 during COVID). This suggests a possible cumulative or lingering effect of stress exposure, where stressors experienced during the pandemic may have had delayed or prolonged impacts on mental health. Results from linear models further supported these findings: both depressive and anxiety symptom severity scores were more strongly associated with negative life stressors in the post-COVID period compared to during COVID. For instance, the adjusted coefficient for PHQ-9 scores was 1.56 post-COVID vs. 0.67 during COVID. These temporal trends may reflect the ongoing socioeconomic consequences of the pandemic, such as financial instability or employment disruptions, which may have persisted or even intensified after the acute public health crisis subsided. The findings underscore the importance of longitudinal and time-sensitive approaches when assessing the psychological effects of major societal disruptions. We used a binary classification of survey responses into “during” and “after” the COVID-19 pandemic, anchored to the lifting of the federal public health emergency in May 2023. While this approach supports interpretability, we acknowledge that pandemic-related stressors evolved over time, and more granular analyses (e.g., by quarter or wave) may better capture nuanced temporal patterns. Future studies could explore trends using finer time intervals to assess shifts in mental health trajectories more precisely.

This study has several limitations. Its cross-sectional design limits the ability to establish causality or temporal relationships between life stressors and mental health outcomes. The reliance on self-reported measures introduces the potential for recall and response biases, which may affect the accuracy of the reported associations. The instruments used to assess life stressors were not previously validated scales; while the items were derived from the MHRC survey to reflect real-world concerns during and after the COVID-19 pandemic, the lack of formal psychometric validation limits the interpretability and generalizability of the stressor measures. Furthermore, the dichotomization of life stressor scores, although implemented to harmonize comparisons and enhance public health relevance, may have oversimplified the complexities of stress exposure. Although each stressor category was based on multiple items and averaged to reflect broader domains (e.g., job-related, financial, COVID-related), collapsing these into binary indicators (<5 vs. ≥ 5) may have obscured important nuances in severity, duration, or cumulative burden. Future research may benefit from preserving the ordinal nature of stress ratings, using continuous scores, or applying advanced methods such as latent class analysis to capture co-occurring patterns of stress exposure. Additionally, although multiple covariates were included in the models, residual confounding by unmeasured variables—such as access to mental health care, social support, or pre-existing psychiatric conditions—cannot be ruled out. In particular, the MHRC dataset did not contain information on participants’ prior mental health diagnoses, which introduces a significant limitation. Pre-existing mental health conditions may both predispose individuals to perceive stressors more negatively and contribute to current mental health symptoms, thereby confounding the observed associations. Without controlling for baseline mental health status, it is difficult to disentangle the extent to which stressors independently contribute to depressive symptoms, anxiety, or suicidal ideation. This limitation reduces the internal validity of our findings and underscores the need for longitudinal studies that account for prior psychiatric history. Moreover, the study did not explicitly adopt a theoretical framework to guide its interpretation of results. While our findings are consistent with stress-symptom associations reported in prior literature, their interpretation would be strengthened by integration with established frameworks such as the diathesis-stress model or conservation of resources (COR) theory. These models could help contextualize why certain individuals may be more vulnerable to stressors than others, or how limited access to psychosocial resources may exacerbate the impact of stress. The absence of theory-driven analysis reflects both the descriptive nature of the MHRC dataset and the need for future work to incorporate theoretical frameworks to guide variable selection and interpretation. The categorization of time periods as “during” and “after” COVID-19 may also not fully capture overlapping or evolving pandemic-related stressors. Additionally, although our focus was on identifying risk factors for poor mental health, the dataset did not include reliable information on protective or buffering factors such as social support, coping strategies, or psychological resilience. This limits our ability to examine potential moderators of stress impact. Inclusion of such variables in future studies would provide a more comprehensive understanding of individual variability in stress responses and could better inform intervention design. Because five specific stressors were modeled within each outcome, we controlled for family‑wise error using Holm adjustment; stressor associations that did not survive Holm are described as positive but non-significant and should be interpreted cautiously. Finally, because the data were collected in Canada, results may not be generalizable to countries with different sociopolitical contexts, public health responses, or healthcare systems. While our study focused on risk factors associated with mental health outcomes, it did not assess protective factors that may buffer against the effects of stress, such as social support, physical activity, or adaptive coping strategies. Prior research highlights the role of resilience in mitigating stress-related psychopathology. Future research should incorporate these resilience-related variables to better understand individual variability in mental health responses to life stressors. Including such factors could provide a more comprehensive and balanced framework for guiding public health interventions. Although multiple polling waves exist, our analytic dataset contained only single complete observations per person and inconsistent identifiers, precluding longitudinal modelling. Future research using harmonised IDs or purpose-built cohorts could examine trajectories and cumulative stress effects over time.

This study highlights the significant associations between specific life stressors and adverse mental health outcomes, including depressive symptoms, anxiety, and suicidal ideation or intent, among Canadian adults during and after the COVID-19 pandemic. Participants who reported negative impacts from challenges such as paying household bills consistently demonstrated higher odds of experiencing these outcomes, with financial stress emerging as the most pervasive factor influencing mental health. Other key stressors, including concerns about catching COVID-19 and economic downturns, also had notable associations, particularly with anxiety and suicidal ideation. The strength of these associations was more pronounced after the pandemic, suggesting a compounding effect of prolonged stressors on mental health. These findings are hypotheses generated for practice and policy. They may inform screening/outreach priorities (e.g., flagging financial strain alongside PHQ‑9/GAD‑7), but the effectiveness of specific interventions was not assessed here and should be evaluated in randomized or quasi‑experimental studies before scale‑up. Addressing economic instability, social isolation, and health-related stressors could be crucial in improving mental health outcomes and fostering resilience during future public health crises. Future studies should build on these findings by employing longitudinal designs to better understand the causal pathways between specific life stressors and mental health outcomes over time. Based on the study findings, it would be valuable to evaluate integrated mental‑health and financial‑assistance programs in Canada. Existing initiatives such as Wellness Together Canada and temporary financial aid programs like the Canada Emergency Response Benefit (CERB) provided critical support during the pandemic but may not be sufficient for long-term recovery. Future efforts should focus on testing whether sustaining and expanding access to comprehensive mental‑health services and targeted financial support improves outcomes, recognizing that the present cross‑sectional data do not estimate intervention effects.

## Supporting information

S1 Table Prevalence of life stressors during and after COVID-19. (DOCX)

S2 Table Associations of life stressors with depressive symptom severity (total PHQ-9 scores) and anxiety severity (total GAD-7 scores). (DOCX)

S3 Table AIC of logistic regression models. (DOCX)

S4 Table Comparison of associations for cases included vs. excluded because of missing covariates (March 2020–January 2024). (DOCX)

S1 Data Data partner memo unique IDs final. (PDF)
