# Supplementary material for: Life stressors and mental health: Depressive symptoms, anxiety, and suicidal ideation or intent during and after the COVID-19 pandemic
Source: PLoS One. 2026 Feb 11;21(2):e0340198. doi: 10.1371/journal.pone.0340198 (PMC12893612; doi:10.1371/journal.pone.0340198)
Supplement: S1 Table — (DOCX) [file pone.0340198.s001.docx]

**Table 1S**. Prevalence of Life Stressors During and After COVID-19.

| **Time** | **Variables** | **Total** | **Depressive Symptoms**  **No** | **Depressive**  **Symptoms**  **Yes** | ***p-value*** | **Moderate to Severe Anxiety**  **No** | **Moderate to Severe Anxiety**  **Yes** | ***p-value*** |
| --- | --- | --- | --- | --- | --- | --- | --- | --- |
| During Covid | **Sample Size** | 14,940 | 11,195 | 3,745 | - | 12,236 | 2,704 | • |
|  | **Life Stressors (%)** |  |  |  | <0.001 |  |  | <0.001 |
|  | Positive Impact | 4,531  (30.8) | 3,639  (33.0) | 892  (23.4) |  | 3,915  (32.6) | 616  (21.7) |  |
|  | Negative Impact | 10,409 (69.2) | 7,556 (67.0) | 2,853   (76.6) |  | 8,321 (67.4) | 2,088  (78.3) |  |
| After Covid | **Sample Size** | 4,985 | 3,666 | 1,319 |  | 3,992 | 993 |  |
|  | **Life Stressors (%)** |  |  |  | <0.001 |  |  | <0.001 |
|  | Positive Impact | 1,642  (33.5) | 1,329  (36.8) | 313  (24.2) |  | 1,408  (35.9) | 234  (23.8) |  |
|  | Negative Impact | 3,343  (66.5) | 2,337  (63.2) | 1,006  (75.8) |  | 2,584  (64.1) | 759  (76.2) |  |

*Anxiety represents “moderate to severe anxiety”. P-values were calculated by weighted Chi-square tests for life stressors, which were reported as raw frequency and weighted percent.
