# Supplementary material for: Life stressors and mental health: Depressive symptoms, anxiety, and suicidal ideation or intent during and after the COVID-19 pandemic
Source: PLoS One. 2026 Feb 11;21(2):e0340198. doi: 10.1371/journal.pone.0340198 (PMC12893612; doi:10.1371/journal.pone.0340198)
Supplement: S2 Table — (DOCX) [file pone.0340198.s002.docx]

**Table 2S**. Associations of Life Stressors with Depressive Symptom Severity (Total PHQ-9 Scores) and Anxiety Severity (Total GAD-7 Scores).

| **Time** | **Life Stressors** | **Depressive Symptom Severity**  **(PHQ-9 scores)** | | | | | **Anxiety Severity**  **(GAD-7 scores)** | | | |  |
| --- | --- | --- | --- | --- | --- | --- | --- | --- | --- | --- | --- |
|  |  | **OR.**  **(95% CI)** | ***p-value*** | **aOR. (95% CI)** | ***p-value*** | ***p-value***  ***(Z-test)*** | **OR.**  **(95% CI)** | ***p-value*** | **aOR. (95% CI)** | ***p-value*** | ***p-value (Z-test)*** |
| March 2020 - Jan 2024 | Positive Impact | • | • | • | • | • | • |  | • | • | • |
|  | Negative Impact | 1.68 (1.48, 1.88) | <0.001 | 1.12  (0.84, 1.40) | <0.001 | • | 1.41 (1.25, 1.58) | <0.001 | 0.94  (0.71, 1.17) | <0.001 | • |
| During Covid | Positive Impact | • | • | • | • | 0.002 | • |  | • | • | 0.004 |
|  | Negative Impact | 1.54  (1.31, 1.78) | <0.001 | 0.67  (0.23, 1.10) | 0.003 |  | 1.27 (1.07, 1.46) | <0.001 | 0.59 (0.2, 0.95) | 0.002 |  |
| After Covid | Positive Impact | • | • | • | • |  | • |  | • | • |  |
|  | Negative Impact | 2.15  (1.77, 2.52) | <0.001 | 1.56  (1.20, 1.92) | <0.001 |  | 1.89 (1.59, 2.20) | <0.001 | 1.29 (0.99, 1.59) | <0.001 |  |
