# Supplementary material for: Life stressors and mental health: Depressive symptoms, anxiety, and suicidal ideation or intent during and after the COVID-19 pandemic
Source: PLoS One. 2026 Feb 11;21(2):e0340198. doi: 10.1371/journal.pone.0340198 (PMC12893612; doi:10.1371/journal.pone.0340198)
Supplement: S3 Table — (DOCX) [file pone.0340198.s003.docx]

**Table 3S**. AIC of Logistic Regression Models.

| **Time** | **Mental Health** | **AIC** |
| --- | --- | --- |
|  |  |  |

| March 2020 - Jan 2024 | Depressive Symptoms | 8,822 |
| --- | --- | --- |
|  | Moderate to Severe Anxiety | 7,586 |
|  | Suicidal Ideation/Intent | 9,612 |
| March 2020 - Jan 2024 | Depressive Symptoms | 8,822 |
|  | Moderate to Severe Anxiety | 7,586 |
| After Covid | Depressive Symptoms | 5,965 |
|  | Moderate to Severe Anxiety | 4,405 |
