# Supplementary material for: Life stressors and mental health: Depressive symptoms, anxiety, and suicidal ideation or intent during and after the COVID-19 pandemic
Source: PLoS One. 2026 Feb 11;21(2):e0340198. doi: 10.1371/journal.pone.0340198 (PMC12893612; doi:10.1371/journal.pone.0340198)
Supplement: S4 Table — (DOCX) [file pone.0340198.s004.docx]

**Table 4S**. Comparison of Associations for Cases Included vs. Excluded Because of Missing Covariates (March 2020–January 2024).

| **Analysis**  **Type** | **Life Stressors** | **Depressive Symptoms** | | **Moderate to Severe Anxiety** | | **Suicidal Ideation/Intent** | |
| --- | --- | --- | --- | --- | --- | --- | --- |
|  |  | **OR**  **(95% CI)** | **p-value** | **OR**  **(95% CI)** | **p-value** | **OR**  **(95% CI)** | **p-value** |
| Included in adjusted model (n = 18,343) | Positive Impact | - | - | - | - | - | - |
|  | Negative Impact | 1.21  (1.06, 1.39) | <0.001 | 1.27  (1.11, 1.46) | <0.001 | 1.58  (1.46, 1.72) | <0.001 |
| Excluded from adjusted model (n = 1,607) | Positive Impact | - | - | - | - | - | - |
|  | Negative Impact | 1.33  (0.83, 2.16) | 0.23 | 1.59  (1.00, 2.51) | 0.04 | 1.49  (1.12, 1.96) | 0.005 |
