# Supplementary material for: Life stressors and mental health: Depressive symptoms, anxiety, and suicidal ideation or intent during and after the COVID-19 pandemic
Source: PLoS One. 2026 Feb 11;21(2):e0340198. doi: 10.1371/journal.pone.0340198 (PMC12893612; doi:10.1371/journal.pone.0340198)
Supplement: S1 Data — (PDF) [file pone.0340198.s005.pdf]

RECHERCHE EN SANTÉ MENTALE CANADA
MENTAL HEALTH RESEARCH CANADA

Our world. Made better.
Through mental health research.

TO: MHRC data partners
DATE: December 15, 2022
SUBJECT: Duplicate Unique Identifiers (UID's)

To our valued data partners:

Recently, MHRC has released our 14th national polling dataset. As we and other partners begin to analyze this data through a longitudinal lens, we wanted to highlight the inconsistencies in the Unique Identifier (Variable "UID") that create challenges in identifying duplicate respondents.

While the bulk of our data continues to be collected by our partner Leger, occasionally additional suppliers are leveraged to collect larger samples within particular provinces (while maintaining methodology consistent with the original data collection). Unfortunately, Leger's Unique Identifier system, intended to ensure data quality, is not a shared system with these additional suppliers. As a result, MHRC cannot confirm the Unique Identifiers (UID's) in data coming from Other Suppliers.

This creates a challenge for our data partners, some of whom are removing duplicate respondents from their datasets. To support these partners, the latest release of our "Merged Polls 0-13" dataset features a new variable "Supplier" (Column YH in your excel file) that identifies whether the data is sourced from Leger (Supplier = 1) or Other Suppliers (Supplier = 2).

As many methodologies exist, how our data partners choose to analyze our datasets is a matter of individual preferences. However, we wanted to highlight this issue and how we are currently addressing it. As such, in any analyses where the intention is to remove duplicate respondents, the data team at MHRC advise duplicate UID values be removed from Leger supplied data only. We do not advise that analysts remove what appear to be duplicate UID's from data sourced from Other Suppliers (Supplier = 2). These have been identified as "false duplicates". (i.e., identical UID, different people).

If you have any questions about our datasets or require advice with cleaning the dataset of duplicate respondents, reach out Shauna Major at smajor@mhrc.ca.

Sincerely,

Mike Cooper

Michael Cooper
Vice President of Mental Health Research Canada
